# Whole Exome-Wide Association Identifies Rare Variants in *APC* Associated with High-Risk Colorectal Cancer in the Middle East

**DOI:** 10.3390/cancers16213720

**Published:** 2024-11-04

**Authors:** Abdul Khalid Siraj, Rong Bu, Saud Azam, Zeeshan Qadri, Kaleem Iqbal, Sandeep Kumar Parvathareddy, Fouad Al-Dayel, Khawla S. Al-Kuraya

**Affiliations:** 1 Human Cancer Genomic Research, King Faisal Specialist Hospital and Research Center, P.O. Box 3354, Riyadh 11211, Saudi Arabia; asiraj@kfshrc.edu.sa (A.K.S.); rbu@kfshrc.edu.sa (R.B.); sjeelani@kfshrc.edu.sa (S.A.); sqadri96@kfshrc.edu.sa (Z.Q.); miqbal@kfshrc.edu.sa (K.I.); psandeepkumar@kfshrc.edu.sa (S.K.P.); 2 Department of Pathology, King Faisal Specialist Hospital and Research Center, P.O. Box 3354, Riyadh 11211, Saudi Arabia; dayelf@kfshrc.edu.sa

**Keywords:** colorectal cancer, exome-wide association study, rare variants, APC, high-risk CRC, Middle East, sequence kernel association test

## Abstract

**Background**: Colorectal cancer (CRC) displays a complex pattern of inheritance. It is postulated that much of the missing heritability of CRC is enriched in high-impact rare alleles, which might play a crucial role in the etiology and susceptibility of CRC. **Methods**: In this study, an exome-wide association analysis was performed in 146 patients with high-risk CRC in the Middle East and 1395 healthy controls. The aim was to identify rare germline variants in coding regions and their splicing sites associated with high-risk CRC in the Middle Eastern population. **Results**: Rare inactivating variants (RIVs) in *APC* had the strongest association with high-risk CRC (6/146 in cases vs. 1/1395 in controls, OR = 59.7, *p* = 5.13 × 10^−12^), whereas RIVs in *RIMS1*, an RAS superfamily member, were significantly associated with high-risk CRC (5/146 case vs. 2/1395 controls, OR = 24.7, *p* = 2.03 × 10^−8^). Rare damaging variants in 17 genes were associated with high-risk CRC at the exome-wide threshold (*p* < 2.5 × 10^−6^). Based on the sequence kernel association test, nonsynonymous variants in six genes (*TNXB*, *TAP2*, *GPSM3*, *ADGRG4*, *TMEM229A*, and *ANKRD33B*) had a significant association with high-risk CRC. RIVs in *APC*—the most common high-penetrance genetic factor—were associated with patients with high-risk CRC in the Middle East. Individuals who inherited *APC* RIVs had an approximate 60-fold increased risk of developing CRC and were likely to develop the disease earlier. **Conclusions**: We identified new potential CRC predisposition variants in other genes that could play a role in CRC inheritance. However, large collaborative studies are needed to confirm the association of these variants with high-risk CRC. These results provide information for counseling patients with high-risk CRC and their families in our population.

## Simple Summary

This study focused on the identification of rare variants that are associated with high-risk colorectal cancer (CRC) from the Middle Eastern region. This study analyzed DNA samples from 146 patients with CRC and from 1395 healthy individuals. We identified rare inactivating variants in the *APC* gene that are strongly linked to CRC, increasing the risk approximately 60-fold. Other significant genes harboring rare damaging variants were also identified. These results may have implications for genetic counseling and the early detection of CRC in the Middle Eastern population.

## 1. Introduction

Colorectal cancer (CRC) is the most frequently diagnosed cancer among males in Saudi Arabia and ranks as the third most common cancer in females, with a median age of 60 years at diagnosis [1]. However, 23% of all cases occur in individuals under the age of 50 [1]. The incidence of CRC in this group is expected to increase because patients aged ≤50 years do not routinely undergo CRC screening [2,3,4].

The genetic landscape of CRC susceptibility spans from highly penetrant germline mutations linked to well-known syndromes to more common polymorphisms. However, in >30% of the cases of familial CRC, the heritable cause is unknown [5,6].

Recent genomic studies have suggested that missing heritability may be attributed to rare, high-impact variants. These rare variants might play crucial roles in human diseases, as evolutionary purifying selection causes damaging alleles to remain rare [7]. Numerous rare variants have been linked to various types of cancer, including thyroid [8], pancreatic [9], and lung [10] cancers as well as CRC [11,12,13,14,15].

Despite the importance of this susceptibility and its clinical relevance to the clinical management of familial CRC, rare variants have not been studied in CRC from Middle Eastern populations in which the incidence of young-onset CRC is higher than that in Western populations.

Therefore, rare variants responsible for CRC susceptibility in Middle Eastern populations must be investigated. Identifying cancer predisposition genes through pathogenic rare variants could provide new insights into the genetic foundations of CRC in Middle Eastern populations. This approach could prove valuable for discovering preventive markers and advancing precision medicine strategies.

In the current study, we utilized whole-exome sequencing (WES) to uncover rare damaging variants (RDVs) and rare inactivating variants (RIVs) linked to high-risk colorectal cancer (CRC) among Middle Eastern populations. The Saudi Cancer Registry indicates that the onset age for colorectal cancer (CRC) is between 58 and 60 years [1], which is significantly younger than the age of 65 years or older reported in Western countries [16]. This earlier onset prompted us to define high-risk individuals in our study as those aged ≤56 years. Consequently, our criteria for identifying high-risk individuals include either ≤56 years or a positive family history of CRC. Using exome data derived from 146 patients with high-risk CRC and 1395 patients without cancer, we confirmed RIVs in *APC*, which is the most prevalent high-penetrance genetic factor linked to high-risk CRC patients in the Middle East. In addition, we identified other candidate variants and/or genes. Our study could facilitate genetic counseling and the tailoring of prevention strategies in these CRC patients.

## 2. Materials and Methods

### 2.1. Patient Selection

Archival samples from 146 patients with high-risk CRC diagnosed between 2000 and 2015 at King Faisal Specialist Hospital and Research Center (KFSHRC) Riyadh, Saudi Arabia, were included in this study. Relaxed criteria were used to select patients at high risk, based on our previous publication [17]. Age ≤ 56 years or a positive family history of cancer were considered high risk. Clinicopathological data were gathered from the medical records of the patients (Table 1). Familial antecedent data for colorectal cancer patients with positive family history (n = 53) has been provided in Supplementary Table S1. This study received approval from the hospital’s Institutional Review Board. As only retrospective patient data were used, the Research Advisory Council (RAC) granted a waiver of consent for the project RAC # 2190 016.

### 2.2. DNA Extraction

DNA was extracted from blood or fresh tissues without tumors using the DNA extraction kit (Gentra, Minneapolis, MN, USA) in accordance with the product protocol, as previously detailed [18]. To ensure the integrity of the DNA from tissues or blood samples, we evaluated its quality using a Nanodrop spectrophotometer and Invitrogen Qubit Fluorometer, ensuring it is suitable for downstream applications.

### 2.3. Whole Exome Sequencing 

WES was conducted for 146 cases using the SureSelectXT Target Enrichment kit (Agilent Technologies, Inc., Santa Clara, CA, USA) on the Illumina sequencing platform, as described previously [8]. GATK and FastQC were utilized to acquire all quality metrics [19]. We performed germline variant calling using GATK’s HaplotypeCaller and variant annotation with ANNOVAR, following the same methodology as outlined in our previous work [8]. This included filtering variants based on minor allele frequency thresholds, quality metrics, and Hardy–Weinberg equilibrium, as well as validating results through the Integrated Genomics Viewer. The control population in our cohort consists of 1395 non-cancer samples from our in-house data gathered from exome sequencing, sequenced at different times. All samples were processed using the same WES methodology. 

RIVs defined as deleterious variants, including stop-loss, stop-gain, and splice-site variants, as well as frameshift insertions and deletions with allele frequencies of less than 0.01, were observed in our control cohort as well as in the Exome Aggregation Consortium (ExAC) database. RDVs are classified as either damaging or inactivating and are predicted to be damaging or pathogenic, exhibiting M-CAP classifier scores of more than 0.025 [9,20]. 

Non-synonymous variants comprised those having allele frequencies below 0.1 in both the ExAC and our cohort. This category included damaging variants, missense variants, as well as non-frameshift insertions and deletions. These variants were utilized in the sequence kernel association test (SKAT) [9]. 

**Figure 1:**
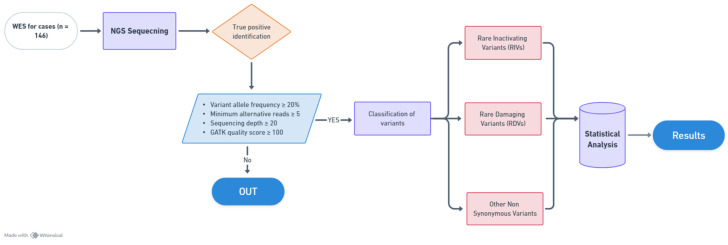
Flowchart illustrating the methodology employed in the study. (Made with Whimsical 2024, online version available at https://whimsical.com).

A Kyoto Encyclopedia of Genes and Genomes (KEGG) pathway analysis was conducted using the Database for Annotation, Visualization and Integrated Discovery (DAVID) platform [21]. The list of genes was analyzed by using default parameters and results were significant if *p* < 0.05. A flowchart illustrating the employed methodology has been presented in Figure 1.

### 2.4. Statistical Analysis

Genes carrying a minimum of five RIVs in the cohort were considered for association analysis. The association of genes between cases and the control population were assessed using χ^2^ test. Associations with a *p*-value of *p* < 2.5 × 10^−6^ were considered to be exome-wide significant, while associations were considered as suggestive if *p* < 0.001 [9]. All statistical tests were conducted as two-sided. We also employed a filter-based methodology for comparison, ranking genes according to the frequency of RIVs present in the cases.

The SKAT method, implemented in the R package, was used to perform the analysis of association. Standard parameters were utilized to determine this association. The *p*-values for nonsynonymous variants were calculated using efficient resampling techniques integrated within the “SKATBinary_Single” algorithm. [9].

## 3. Results

The participants in the study had an average age of 41 years, with slightly more males than females. Most cases were adenocarcinoma, while a smaller fraction of cases were mucinous CRC. A significant number of tumors were found to be moderately differentiated and primarily located on the left side. Detailed clinicopathological characteristics have been mentioned in Table 1. 

We identified a total of 266,030 variants across 17,300 genes, which included 218,056 rare damaging variants (24,010 in cases and 194,046 in controls) and 17,040 rare inactivating variants (1585 in cases and 15,455 in controls). The median number of RIVs was 10 (interquartile range: 6–14) in cases and 11 (interquartile range: 7–14) in controls, respectively (*p* = 0.510 by Wilcoxon rank sum test).

Our primary analysis was focused on RIVs, where the strongest association was observed in *APC* with an approximate 60-fold increased risk of developing high-risk CRC (odds ratio adjusted OR = 59.7, *p* = 5.08 × 10^−8^), meeting exome-wide significance (*p* < 2.5 × 10^−6^). In total, 6/146 (4.1%) cases carried RIVs compared with 1/1395 (0.1%) controls. This association was driven by five variants as follows: chr5: 112154963C>T, the most common missense variant, was observed in 3/146 cases (2.1%) and 0/1395 controls (OR = 68.1, *p* = 8.36 × 10^−8^); three other variants were identified in one case each and were not present in the controls (OR = 28.8, *p* = 0.002) (Table 2); and the last variant, a frameshift deletion of chr5:112175077Tdel, was detected in one control but was absent in cases (OR = 0.31, *p* = 0.746).

In addition to *APC*, *RIMS1*, an RAS superfamily member, passed the exome-wide significance threshold and was the second most significant gene with a ~24-fold increased risk of developing high-risk CRC (OR = 24.7, *p* = 2.03 × 10^−8^) when comparing cases with controls (Table 2). *RIMS1* was positive in 5/146 (3.4%) vs. 2/1395 controls (0.1%). The most significant variant in *RIMS1* was chr6: 72974704T>G, a missense variant observed in 4/146 cases (2.7%) and 0/1395 controls (OR = 88.1, *p* = 6.01 × 10^−10^) (Table 2). 

At the suggested threshold, RIVs in ST6 *N*-acetylgalactosaminide alpha-2, 6-sialyltransferase 2 (*ST6GALNAC2*) were found to be significant (OR = 14.6, *p* = 1.12 × 10^−4^), observed in 3/146 cases (2%), with a 14-fold increased risk of developing high-risk CRC, compared with 2/1395 controls (0.1%) (Table 2). Two cases (1.4%) carried a frameshift deletion (chr17: 74566661del) compared with 1/1395 controls (0.1%) (OR = 19.4, *p* = 0.001) (Table 2).

Furthermore, we used a filter-based method to examine 10 genes carrying the highest number of RIVs in cases, determined by counting. Out of these 10 genes, APC was ranked at the top with six RIVs, whereas the lowest number was found in *ITGA10* with two RIVs. When comparing cases vs. controls, five genes showed significant associations (*p* < 0.05), including *APC* (*p* = 5.08 × 10^−12^), *RIMS1* (*p* = 2.03 × 10^−8^), *ACOT4* (*p* = 0.003), *ST6GALNAC2* (*p* = 1.12 × 10^−4^), and *FSIP2* (*p* = 0.026) (Table 3).

A KEGG pathway analysis was conducted to evaluate the signaling pathways. However, no significant association was observed for any pathways with an elevated risk of CRC development.

We also concentrated on RDVs in both cases and controls for the secondary analysis. A total of 17 genes achieved exome-wide significance (Table 4). The associations were driven by multiple RDVs, and 14 variants were identified among two or three individuals. The highest association was observed for *SPRED1* chr15: 38545392C>A, *SHANK1* chr19:51206940G>A, *OR5K4* chr3: 98073028G>T, and *COL11A2* chr6: 33154366G>T variants present in 3/146 cases (2.1%) each and absent in controls (OR = 68.1 *p* = 8.36 × 10^−8^) (Supplementary Table S2). Interestingly, on comparing the clinicopathological characteristics of these three cases, we found that 66.7% (2/3) were female and all three cases had lymph node metastasis, with one of the patients also exhibiting distant metastasis involving the liver. Two of the three patients had tumor in the left colon, while the other patient had right colon tumor. In addition, there were no other germline pathogenic variants in the cancer-related genes identified in these cases.

At the suggestive threshold (*p* < 0.001), we found RDVs in 117 genes significantly associated with high-risk CRC (Supplementary Table S3). 

For tertiary analyses, we utilized SKAT to assess the combined impact of all non-synonymous variants having cohort allele frequency <0.1. It was observed that seven genes had exome-wide significant associations as follows: tenascin XB (*TNXB*); transporter 2; ATP binding cassette subfamily B member (*TAP2*); G protein signaling modulator 3 (*GPSM3*); adhesion G protein-coupled receptor G4 (*ADGRG4*); transmembrane protein 229A (*TMEM229A*); and ankyrin repeat domain 33B (*ANKRD33B*) (Table 5). Each gene contained a minimum of one variant having *p* < 0.001. Three variants showed significant associations at the exome-wide threshold after Bonferroni’s correction (Supplementary Table S4).

## 4. Discussion

New strategies to prevent CRC are urgently needed, especially in understudied ethnicities, such as Middle Eastern populations. Identifying inherited, rare germline genetic variants in this ethnicity that increase the risk of CRC may improve prevention strategies, helping to reduce the high incidence of high-risk CRC.

Next-generation sequencing has opened new avenues for exploring the genetic causes of cancer and has added new unbiased approaches to facilitate the identification of new genes and/or variants responsible for predisposition to human disease.

In this study, we conducted WES to identify novel rare variants and genes associated with CRC susceptibility, potentially explaining the high-risk of CRC observed in these patients. For this purpose, we analyzed germline data from 146 unrelated cases diagnosed with high-risk CRC and 1395 cancer-free controls. The analysis focused exclusively on patients from the Middle Eastern population, with stringent quality control measures implemented to ensure data integrity.

In the primary analysis, we concentrated on RIVs. *APC* demonstrated the most significant association with an approximate 60-fold increased risk of developing high-risk CRC, meeting the exome-wide threshold. Our primary analyses led to the identification of four RIVs in *APC* in six cases, with a significant association with high-risk CRC in our research population. 

Interestingly, our analysis detected one frameshift deletion in one control, but it was absent in cases, contrary to reports that deleterious variants in *APC* increased the risk of CRC. One explanation might be the limited sample size in this study. Therefore, large-scale studies must be used to investigate the role of this deleterious variant in the development of high-risk CRC in our population.

Patients with germline pathogenic variants in the *APC* gene develop multiple adenomatous polyps in their colon ranging from tens in attenuated familial polyposis [AFAP]) to countless in classic FAP, which significantly elevates their lifetime risk of developing CRC [22,23,24]. It is not surprising that *APC* emerged as the most interesting gene in our analysis. Hence, the role of *APC* pathogenic germline mutations in CRC susceptibility was firmly established, with a prevalence of 5–18% [25,26,27,28].

In our primary analyses, RIVs in *RIMS1* were significantly associated with an approximate 25-fold increased risk of developing high-risk CRC. *RIMS1* is an RAS superfamily member. It is one of the genes that is significantly downregulated in the classical multidrug resistance gastric carcinoma cell line [29]. *RIMS1* mutations impacted survival in patients with pancreatic cancer [30] and gastric cancer [31]. Recently, mutations in *RIMS1* were identified as a potential causal mutation in Chinese familial hemangioblastoma [32].

The enrichment of RDVs in the other 17 genes further bolsters our insight of the inherited genetic basis of colorectal cancer. All of these 17 genes, except *COL11A2*, was reported to be associated with several types of cancer, such as ovarian, gastrointestinal, lung, and pancreatic cancers [33,34,35,36,37,38,39,40,41,42,43,44,45,46,47,48,49]. However, studies on the roles of *COL11A2* in the progression of cancer are lacking. 

We attempted to combine the rare variants to pathways throughout the genome to explore the associations with increased CRC risk through KEGG pathway analysis. No signal pathways were found to be significantly linked to CRC. 

A limitation of the study is the inclusion of cases from a specific population, which precludes the applicability of the results to the general population. Despite this limitation, our results using WES data provide valuable insights into deleterious and disruptive rare coding variants and susceptibility genes for CRC cancer risk in Middle Eastern populations.

Future studies with larger sample sizes and collaborative multicenter samples could allow deep targeted sequencing to reveal other promising variants and additional disease- susceptible genes for CRC in Middle Eastern populations.

## 5. Conclusions

The current study utilized whole-exome sequencing to identify rare variants linked to high-risk CRC. These findings provide important insights into the genetic foundations of CRC in this understudied demographic. The identification of these susceptibility variants may inform the development of targeted prevention strategies, potentially reducing the burden of CRC in Middle Eastern communities. Future large sample studies with broader geographic representation are necessary to further elucidate the genetic landscape of CRC in this region and to refine targeted prevention approaches.

## Figures and Tables

**Table 1:** Clinicopathological characteristics of the study cohort.

	N = 146
**Age (years)**	
Median (IQR), years	41.0 (34.0–48.9)
<50	119 (81.5)
≥50	27 (18.5)
**Gender**	
Female	64 (43.8)
Male	82 (56.2)
**Family history of cancer**	
Positive	53 (36.3)
Negative	93 (63.7)
**Family history of colon cancer**	
Positive	28 (19.2)
Negative	118 (80.8)
**Body mass index (kg/m^2^)**	
<30	92 (63.0)
≥30	40 (27.4)
Unknown	14 (9.6)
**History of diabetes mellitus**	
Present	19 (13.0)
Absent	97 (66.4)
Unknown	30 (20.5)
**Histologic subtype**	
Adenocarcinoma	127 (87.0)
Mucinous	19 (13.0)
**Tumor location**	
Left colon	113 (77.4)
Right colon	26 (17.8)
Transverse colon	7 (4.8)
**Histologic grade**	
Well differentiated	8 (5.5)
Moderately differentiated	113 (77.4)
Poorly differentiated	14 (9.6)
Unknown	11 (7.5)
**pT**	
T1	4 (2.8)
T2	17 (11.7)
T3	90 (61.6)
T4	24 (16.4)
Unknown	11 (7.5)
**pN**	
N0	56 (38.4)
N1/N2	79 (54.1)
Nx	11 (7.5)
**pM**	
M0	111 (76.0)
M1	28 (19.2)
Mx	7 (4.8)
**TNM Stage**	
I	17 (11.7)
II	38 (26.0)
III	55 (37.7)
IV	28 (19.2)
Unknown	8 (5.4)

**Table 2:** List of rare inactivating variants associated with CRC risk.

S No	Gene	Chr	Position	Ref	Alt	Variant Type	No. of Cases	%	No. of Controls	%	*p*-Value	Odds Ratio
1	*APC*	chr5	112,154,963	C	T	Missense	3	2.1	0	0.0	8.36 × 10^−8^	68.1
2	*APC*	chr5	112,128,191	C	T	Missense	1	0.7	0	0.0	0.002	28.8
3	*APC*	chr5	112,155,042	G	C	Missense	1	0.7	0	0.0	0.002	28.8
4	*APC*	chr5	112,174,112	G	T	Missense	1	0.7	0	0.0	0.002	28.8
5	*APC*	chr5	112,175,077	T	-	Frameshift Deletion	0	0.7	1	0.0	0.746	−3.1
6	*RIMS1*	chr6	72,974,704	T	G	Missense	4	2.7	0	0.0	6.01 × 10^−10^	88.1
7	*RIMS1*	chr6	72,975,696	-	TC	Frameshift Insertion	1	0.7	0	0.0	0.002	28.8
8	*RIMS1*	chr6	72,945,397	T	C	Missense	0	0.0	1	0.1	0.746	3.2
9	*RIMS1*	chr6	72,984,083	C	T	Missense	0	0.0	1	0.1	0.746	3.2
10	*ST6GALNAC2*	chr17	74,566,661	T	-	Frameshift Deletion	2	1.4	1	0.1	0.001	19.4
11	*ST6GALNAC2*	chr17	74,568,782	G	-	Frameshift Deletion	1	0.7	1	0.1	0.050	9.6

**Table 3:** List of top 10 genes by number of cases in RIVs.

S No	Gene	No. of Cases	% Cases	No. of Controls	% Controls	*p*-Value	Odds Ratio
1	APC *	6	4.1	1	0.1	5.08 × 10^−12^	59.7
2	*CD36*	6	4.1	25	1.8	0.058	2.3
3	RIMS1 *	5	3.4	2	0.1	2.03 × 10^−8^	24.7
4	*ACOT4*	3	2.1	4	0.3	0.003	7.3
5	*ST6GALNAC2 ^#^*	3	2.1	2	0.1	1.12 × 10^−4^	14.6
6	*FSIP2*	3	2.1	7	0.5	0.026	4.2
7	*PNPLA7*	3	2.1	9	0.6	0.065	3.2
8	*TTN*	2	1.4	32	2.3	0.470	0.6
9	*TTLL10*	2	1.4	5	0.4	0.084	3.9
10	*ITGA10*	2	1.4	52	3.7	0.140	0.4

* Genes passing exome-wide significance level (*p* < 2.5 *×* 10^−6^*)*. ^#^ Gene passing suggestive significance level (*p* < 0.001).

**Table 4:** List of genes with significant RDVs at the exome-wide level (*p* < 2.5 × 10^−6^).

S No	Gene	No. of Cases	% Cases	No. of Controls	% Controls	*p*-Value	Odds Ratio
1	*TNXB*	10	6.8	1	0.1	0.00 × 10^−0^	102.5
2	*GPR112*	7	4.8	0	0.0	0.00 × 10^−0^	150.1
3	*COL11A2*	5	3.4	0	0.0	4.42 × 10^−12^	108.5
4	*ANKRD33B*	4	2.7	1	0.1	6.91 × 10^−8^	39.3
5	*TBKBP1*	5	3.4	2	0.1	2.03 × 10^−8^	24.7
6	*OR5K4*	5	3.4	2	0.1	2.03 × 10^−8^	24.7
7	*MTAP*	5	3.4	2	0.1	2.03 × 10^−8^	24.7
8	*SHANK1*	10	6.8	13	0.9	2.02 × 10^−8^	7.8
9	*SPRED1*	4	2.7	1	0.1	6.91 × 10^−8^	39.3
10	*MPP2*	4	2.7	1	0.1	6.91 × 10^−8^	39.3
11	*KANSL1*	4	2.7	1	0.1	6.91 × 10^−8^	39.3
12	*PIAS4*	4	2.7	1	0.1	6.91 × 10^−8^	39.3
13	*TNNI3*	4	2.7	1	0.1	6.91 × 10^−8^	39.3
14	*BTNL2*	4	2.7	1	0.1	6.91 × 10^−8^	39.3
15	*CAPZA1*	4	2.7	2	0.1	1.64 × 10^−6^	19.6
16	OSTC	4	2.7	2	0.1	1.64 × 10^−6^	19.6
17	SOX4	4	2.7	2	0.1	1.64 × 10^−6^	19.6

**Table 5:** List of genes significant at the exome-wide level analyzed by SKAT.

S No	Gene	No. of Cases	% Cases	No. of Controls	% Controls	*p*-Value	Odds Ratio
1	*TNXB*	16	11.0	1	0.1	0.00 × 10^−0^	171.6
2	*TAP2*	8	5.5	0	0.0	0.00 × 10^−0^	171.3
3	*GPSM3*	7	4.8	0	0.0	0.00 × 10^−0^	150.1
4	*ADGRG4*	7	4.8	0	0.0	0.00 × 10^−0^	150.1
5	*TMEM229A*	8	5.5	4	0.3	1.11 × 10^−11^	20.2
6	*ANKRD33B*	6	4.1	1	0.1	5.08 × 10^−12^	59.7

## Data Availability

The original contributions presented in the study are included in the article/Supplementary Materials. Further inquiries can be directed to the corresponding author.

## Supplementary Materials

The following supporting information can be downloaded at https://www.mdpi.com/article/10.3390/cancers16213720/s1, Table S1: Familial antecedent data for colorectal cancer patients with positive family history (n = 53); Table S2: List of rare damaging variants associations with CRC risk; Table S3: List of RDV genes significant at the suggestive level (*p* < 0.001); Table S4: List of variants significantly related to CRC risk in seven SKAT genes.
