# Supplementary material for: Whole Exome-Wide Association Identifies Rare Variants in APC Associated with High-Risk Colorectal Cancer in the Middle East
Source: Cancers (Basel). 2024 Nov 4;16(21):3720. doi: 10.3390/cancers16213720 (PMC11545597; doi:10.3390/cancers16213720)
Supplement: Supplementary file 1 [file cancers-16-03720-s001.zip › cancers-3252808-supplementary.pdf]

cancers MDPI

Supplementary Table S1: Familial antecedent data for colorectal cancer patients with positive family history (n = 53).

S. No.	Affected family member	Type of cancer in family member
1	Father, Brother and Sister	Colorectal cancer
2	Father and two Sisters	Colorectal cancer
3	Sister	Colorectal cancer
4	Grandmother	Colorectal cancer
5	Brother	Colorectal cancer
6	Father and Sister	Colorectal cancer
7	Father	Hepatocellular carcinoma
8	Father and Brother	Colorectal cancer
9	Father and Sister	Colorectal cancer
10	Sister	Colorectal cancer
11	Maternal Uncles, Sister, Mother	Colorectal cancer
12	Brother<br>Sister	Hodgkin lymphoma<br>Hepatocellular carcinoma
13	Sister	Colorectal cancer
14	Father	Pancreatic cancer
15	Siblings (gender not specified)	Colorectal cancer and Brain tumor (type not specified)
16	Uncle	Colorectal cancer
17	Father, Grandfather and Uncle	Colorectal cancer
18	Mother<br>Brother	Breast cancer<br>Colorectal cancer
19	Grandmother and Uncle	Type of cancer not mentioned
20	Father, Grandfather and Uncles	Type of cancer not mentioned
21	Two Sisters<br>Brother	Leukemia<br>Brain tumor (type not specified)
22	Father	Spinal cord tumor (type not specified)
23	Father and Brother	Type of cancer not mentioned
24	Two Uncles and Grandfather	Brain tumor (type not specified)
25	Grandfather<br>Grandmother	Hepatocellular carcinoma<br>Pancreatic cancer
26	Three Sisters	Breast Cancer
27	Mother<br>Aunt	Endometrial Cancer<br>Breast Cancer
28	Father<br>Two Brothers	Gallbladder cancer<br>Colorectal cancer
29	Relationship not mentioned	Colorectal cancer
30	Aunt	Hepatocellular carcinoma
31	Father	Colorectal cancer

32	Daughter<br>Paternal Cousin	Breast Cancer<br>Renal cell carcinoma
33	Two Uncles	Colorectal cancer
34	Brother and Father<br>Sister	Colorectal cancer<br>Endometrial Cancer
35	Sister	Colorectal cancer
36	Son and Daughter	Colorectal cancer
37	Aunt and Grandmother	Cervix Cancer
38	Paternal Grandmother<br>Maternal cousin	Brain tumor (type not specified)<br>Colorectal cancer
39	Mother<br>Maternal Grandmother<br>Paternal uncle	Pancreatic cancer<br>Colorectal cancer<br>Lung cancer
40	Maternal Grandfather	Prostate cancer
41	Maternal Grandmother	Breast cancer
42	Paternal Uncle<br>Paternal Aunt	Renal cell carcinoma<br>Sarcoma (not specified)
43	Paternal Uncle	Gastric cancer
44	Grandmother<br>Maternal Aunt	Gastric cancer<br>Breast cancer
45	Father	Hepatocellular carcinoma
46	Grandfather	Type of cancer not mentioned
47	Grandmother<br>Uncle<br>Aunt<br>Cousin	Lung cancer<br>Colorectal cancer<br>Hepatocellular carcinoma<br>Esophageal cancer
48	Uncle	Lung cancer
49	Aunt	Breast cancer
50	Paternal Uncle<br>First Paternal Cousin<br>Second Paternal Cousin	Colorectal cancer<br>Laryngeal cancer<br>Leukemia
51	Maternal grandmother	Breast cancer
52	Brother	Colorectal cancer
53	3rd Degree	Colorectal cancer

Supplementary Table S2: List of rare damaging variants associations with CRC risk.

S No	Gene	Chr	Position	Ref	Alt	No. of Cases	%	No. of Controls	%	P-value	Odds ratio
1.	*SPRED1*	chr15	38,545,392	C	A	3	2.1	0	0.0	8.36×10^{-08}	68.1
2.	*SHANK1*	chr19	51,206,940	G	A	3	2.1	0	0.0	8.36×10^{-08}	68.1
3.	*OR5K4*	chr3	98,073,028	G	T	3	2.1	0	0.0	8.36×10^{-08}	68.1
4.	*COL11A2*	chr6	33,154,366	G	T	3	2.1	0	0.0	8.36×10^{-08}	68.1
5.	*CAPZA1*	chr1	113,162,500	G	A	3	2.1	1	0.1	7.44×10^{-06}	29.2
6.	*KANSL1*	chr17	44,172,062	C	A	2	1.4	0	0.0	1.22×10^{-05}	48.3
7.	*PIAS4*	chr19	4,013,263	G	A	2	1.4	0	0.0	1.22×10^{-05}	48.3
8.	*ANKRD33B*	chr5	10,638,251	C	T	2	1.4	0	0.0	1.22×10^{-05}	48.3
9.	*SOX4*	chr6	21,595,190	G	A	2	1.4	0	0.0	1.22×10^{-05}	48.3
10.	*TNXB*	chr6	32,049,429	G	T	2	1.4	0	0.0	1.22×10^{-05}	48.3
11.	*BTNL2*	chr6	32,362,627	G	T	2	1.4	0	0.0	1.22×10^{-05}	48.3
12.	*MTAP*	chr9	21,818,137	T	G	2	1.4	0	0.0	1.22×10^{-05}	48.3
13.	*MTAP*	chr9	21,854,630	G	A	2	1.4	0	0.0	1.22×10^{-05}	48.3
14.	*TNNI3*	chr19	55,666,125	G	T	2	1.4	1	0.1	7.10×10^{-04}	19.4
15.	*CAPZA1*	chr1	113,197,201	G	A	1	0.7	0	0.0	0.002	28.8
16.	*SPRED1*	chr15	38,614,584	G	A	1	0.7	0	0.0	0.002	28.8
17.	*MPP2*	chr17	41,958,884	C	T	1	0.7	0	0.0	0.002	28.8
18.	*MPP2*	chr17	41,959,837	G	A	1	0.7	0	0.0	0.002	28.8
19.	*MPP2*	chr17	41,960,609	C	T	1	0.7	0	0.0	0.002	28.8
20.	*MPP2*	chr17	41,986,921	C	T	1	0.7	0	0.0	0.002	28.8
21.	*KANSL1*	chr17	44,248,233	C	G	1	0.7	0	0.0	0.002	28.8
22.	*KANSL1*	chr17	44,248,791	G	A	1	0.7	0	0.0	0.002	28.8
23.	*TBKBP1*	chr17	45,773,551	G	A	1	0.7	0	0.0	0.002	28.8
24.	*TBKBP1*	chr17	45,776,016	G	A	1	0.7	0	0.0	0.002	28.8
25.	*TBKBP1*	chr17	45,776,854	G	A	1	0.7	0	0.0	0.002	28.8
26.	*TBKBP1*	chr17	45,786,111	T	A	1	0.7	0	0.0	0.002	28.8
27.	*TBKBP1*	chr17	45,786,525	C	T	1	0.7	0	0.0	0.002	28.8
28.	*PIAS4*	chr19	4,013,123	C	T	1	0.7	0	0.0	0.002	28.8
29.	*PIAS4*	chr19	4,037,629	A	G	1	0.7	0	0.0	0.002	28.8
30.	*SHANK1*	chr19	51,165,335	C	T	1	0.7	0	0.0	0.002	28.8
31.	*SHANK1*	chr19	51,165,685	T	C	1	0.7	0	0.0	0.002	28.8
32.	*SHANK1*	chr19	51,165,821	C	T	1	0.7	0	0.0	0.002	28.8
33.	*SHANK1*	chr19	51,170,394	G	C	1	0.7	0	0.0	0.002	28.8
34.	*SHANK1*	chr19	51,170,521	G	A	1	0.7	0	0.0	0.002	28.8
35.	*SHANK1*	chr19	51,171,273	G	A	1	0.7	0	0.0	0.002	28.8
36.	*SHANK1*	chr19	51,192,145	C	T	1	0.7	0	0.0	0.002	28.8
37.	*TNNI3*	chr19	55,663,245	G	A	1	0.7	0	0.0	0.002	28.8
38.	*TNNI3*	chr19	55,665,519	G	T	1	0.7	0	0.0	0.002	28.8
39.	*OR5K4*	chr3	98,073,030	C	G	1	0.7	0	0.0	0.002	28.8
40.	*OR5K4*	chr3	98,073,061	C	T	1	0.7	0	0.0	0.002	28.8
41.	*OSTC*	chr4	109,571,907	TG	-	1	0.7	0	0.0	0.002	28.8
42.	*OSTC*	chr4	109,578,797	A	C	1	0.7	0	0.0	0.002	28.8
43.	*OSTC*	chr4	109,584,430	T	C	1	0.7	0	0.0	0.002	28.8

44.	*OSTC*	chr4	109,588,403	G	A	1	0.7	0	0.0	0.002	28.8
45.	*ANKRD33B*	chr5	10,564,604	C	T	1	0.7	0	0.0	0.002	28.8
46.	*ANKRD33B*	chr5	10,564,904	G	C	1	0.7	0	0.0	0.002	28.8
47.	*ANKRD33B*	chr5	10,649,618	C	T	1	0.7	0	0.0	0.002	28.8
48.	*SOX4*	chr6	21,595,639	A	G	1	0.7	0	0.0	0.002	28.8
49.	*SOX4*	chr6	21,595,640	A	T	1	0.7	0	0.0	0.002	28.8
50.	*SOX4*	chr6	21,595,877	C	G	1	0.7	0	0.0	0.002	28.8
51.	*TNXB*	chr6	32,020,588	G	A	1	0.7	0	0.0	0.002	28.8
52.	*TNXB*	chr6	32,030,189	G	A	1	0.7	0	0.0	0.002	28.8
53.	*TNXB*	chr6	32,041,457	G	A	1	0.7	0	0.0	0.002	28.8
54.	*TNXB*	chr6	32,041,679	G	A	1	0.7	0	0.0	0.002	28.8
55.	*TNXB*	chr6	32,049,219	C	T	1	0.7	0	0.0	0.002	28.8
56.	*TNXB*	chr6	32,064,047	C	T	1	0.7	0	0.0	0.002	28.8
57.	*TNXB*	chr6	32,064,464	A	C	1	0.7	0	0.0	0.002	28.8
58.	*TNXB*	chr6	32,065,005	T	C	1	0.7	0	0.0	0.002	28.8
59.	*BTNL2*	chr6	32,362,551	C	T	1	0.7	0	0.0	0.002	28.8
60.	*BTNL2*	chr6	32,364,044	G	A	1	0.7	0	0.0	0.002	28.8
61.	*BTNL2*	chr6	32,373,064	C	T	1	0.7	0	0.0	0.002	28.8
62.	*COL11A2*	chr6	33,154,514	C	A	1	0.7	0	0.0	0.002	28.8
63.	*COL11A2*	chr6	33,157,099	G	T	1	0.7	0	0.0	0.002	28.8
64.	*MTAP*	chr9	21,854,717	C	G	1	0.7	0	0.0	0.002	28.8
65.	*GPR112*	chrX	135,390,947	A	G	1	0.7	0	0.0	0.002	28.8
66.	*GPR112*	chrX	135,405,191	T	C	1	0.7	0	0.0	0.002	28.8
67.	*GPR112*	chrX	135,405,471	A	G	1	0.7	0	0.0	0.002	28.8
68.	*GPR112*	chrX	135,428,194	A	T	1	0.7	0	0.0	0.002	28.8
69.	*GPR112*	chrX	135,431,195	C	A	1	0.7	0	0.0	0.002	28.8
70.	*GPR112*	chrX	135,431,514	G	A	1	0.7	0	0.0	0.002	28.8
71.	*GPR112*	chrX	135,441,498	C	A	1	0.7	0	0.0	0.002	28.8
72.	*OSTC*	chr4	109,584,429	G	A	1	0.7	1	0.1	0.050	9.6

Supplementary Table S3: List of RDV genes significant at the suggestive level (*P* < 0.001).

S No	Gene	No. of Cases	% Cases	No. of Controls	% Controls	p_value	Odds ratio
1	*DDI2*	7	4.8	9	0.6	2.53×10^{-06}	7.8
2	*CBFA2T3*	7	4.8	9	0.6	2.53×10^{-06}	7.8
3	*MMP25*	7	4.8	10	0.7	7.19×10^{-06}	7.0
4	*ZNF729*	5	3.4	5	0.4	1.13×10^{-05}	9.9
5	*ZNF787*	5	3.4	5	0.4	1.13×10^{-05}	9.9
6	*SEMA5B*	5	3.4	5	0.4	1.13×10^{-05}	9.9
7	*COL7A1*	16	11.0	48	3.4	1.48×10^{-05}	3.5
8	*HPN*	4	2.7	3	0.2	1.59×10^{-05}	13.1
9	*MAN1A1*	4	2.7	3	0.2	1.59×10^{-05}	13.1
10	*SLC35B2*	4	2.7	3	0.2	1.59×10^{-05}	13.1
11	*AGK*	7	4.8	11	0.8	1.82×10^{-05}	6.3
12	*PERM1*	6	4.1	8	0.6	1.83×10^{-05}	7.4
13	*TLE6*	6	4.1	8	0.6	1.83×10^{-05}	7.4
14	*CD207*	6	4.1	8	0.6	1.83×10^{-05}	7.4
15	*HTR3C*	6	4.1	8	0.6	1.83×10^{-05}	7.4
16	*NACAD*	8	5.5	15	1.1	2.97×10^{-05}	5.3
17	*DIP2A*	10	6.8	23	1.6	3.63×10^{-05}	4.4
18	*PLCD1*	7	4.8	12	0.9	4.15×10^{-05}	5.8
19	*TROVE2*	5	3.4	6	0.4	4.33×10^{-05}	8.2
20	*COL4A3BP*	5	3.4	6	0.4	4.33×10^{-05}	8.2
21	*PREP*	5	3.4	6	0.4	4.33×10^{-05}	8.2
22	*KHDC3L*	5	3.4	6	0.4	4.33×10^{-05}	8.2
23	*ADAM7*	5	3.4	6	0.4	4.33×10^{-05}	8.2
24	*SLC38A2*	6	4.1	9	0.6	4.98×10^{-05}	6.6
25	*FAM181B*	4	2.7	4	0.3	8.70×10^{-05}	9.8
26	*RBM26*	4	2.7	4	0.3	8.70×10^{-05}	9.8
27	*TIGD5*	4	2.7	4	0.3	8.70×10^{-05}	9.8
28	*CCDC153*	8	5.5	17	1.2	1.06×10^{-04}	4.7
29	*SNX27*	3	2.1	2	0.1	1.12×10^{-04}	14.6
30	*RRM1*	3	2.1	2	0.1	1.12×10^{-04}	14.6
31	*FAU*	3	2.1	2	0.1	1.12×10^{-04}	14.6
32	*HOXC13*	3	2.1	2	0.1	1.12×10^{-04}	14.6
33	*ULK3*	3	2.1	2	0.1	1.12×10^{-04}	14.6
34	*PEAK1*	3	2.1	2	0.1	1.12×10^{-04}	14.6
35	*METTL26*	3	2.1	2	0.1	1.12×10^{-04}	14.6
36	*FUT1*	3	2.1	2	0.1	1.12×10^{-04}	14.6
37	*BAX*	3	2.1	2	0.1	1.12×10^{-04}	14.6
38	*C19orf84*	3	2.1	2	0.1	1.12×10^{-04}	14.6
39	*SDC1*	3	2.1	2	0.1	1.12×10^{-04}	14.6
40	*PROKR1*	3	2.1	2	0.1	1.12×10^{-04}	14.6
41	*PCBP3*	3	2.1	2	0.1	1.12×10^{-04}	14.6
42	*CTBP2*	6	4.1	10	0.7	1.19×10^{-04}	5.9
43	*TOX2*	6	4.1	10	0.7	1.19×10^{-04}	5.9
44	*TTYH3*	6	4.1	10	0.7	1.19×10^{-04}	5.9

45	*PPIE*	5	3.4	7	0.5	1.32×10^{-04}	7.0
46	*SLC25A39*	5	3.4	7	0.5	1.32×10^{-04}	7.0
47	*CDKAL1*	5	3.4	7	0.5	1.32×10^{-04}	7.0
48	*TFR2*	5	3.4	7	0.5	1.32×10^{-04}	7.0
49	*PTK2B*	6	4.1	11	0.8	2.57×10^{-04}	5.4
50	*PODN*	6	4.1	11	0.8	2.57×10^{-04}	5.4
51	*COG6*	6	4.1	11	0.8	2.57×10^{-04}	5.4
52	*NEFH*	6	4.1	11	0.8	2.57×10^{-04}	5.4
53	*GCAT*	7	4.8	15	1.1	3.13×10^{-04}	4.6
54	*ACBD7*	4	2.7	5	0.4	3.27×10^{-04}	7.8
55	*HDAC7*	4	2.7	5	0.4	3.27×10^{-04}	7.8
56	*LDHD*	4	2.7	5	0.4	3.27×10^{-04}	7.8
57	*LMTK3*	4	2.7	5	0.4	3.27×10^{-04}	7.8
58	*PTBP1*	4	2.7	5	0.4	3.27×10^{-04}	7.8
59	*DDX56*	4	2.7	5	0.4	3.27×10^{-04}	7.8
60	*NUTM1*	5	3.4	8	0.6	3.38×10^{-04}	6.1
61	*USP8*	5	3.4	8	0.6	3.38×10^{-04}	6.1
62	*LMOD3*	5	3.4	8	0.6	3.38×10^{-04}	6.1
63	*TLR3*	5	3.4	8	0.6	3.38×10^{-04}	6.1
64	*RUFY1*	5	3.4	8	0.6	3.38×10^{-04}	6.1
65	*LIPE*	9	6.2	24	1.7	4.17×10^{-04}	3.8
66	*NEO1*	10	6.8	29	2.1	4.80×10^{-04}	3.5
67	*ARHGEF11*	7	4.8	16	1.1	5.44×10^{-04}	4.3
68	*CEPT1*	3	2.1	3	0.2	6.83×10^{-04}	9.7
69	*ADIPOR1*	3	2.1	3	0.2	6.83×10^{-04}	9.7
70	*MRGPRG*	3	2.1	3	0.2	6.83×10^{-04}	9.7
71	*OR52K1*	3	2.1	3	0.2	6.83×10^{-04}	9.7
72	*PIANP*	3	2.1	3	0.2	6.83×10^{-04}	9.7
73	*NECAP1*	3	2.1	3	0.2	6.83×10^{-04}	9.7
74	*TM9SF1*	3	2.1	3	0.2	6.83×10^{-04}	9.7
75	*CDYL2*	3	2.1	3	0.2	6.83×10^{-04}	9.7
76	*PCTP*	3	2.1	3	0.2	6.83×10^{-04}	9.7
77	*CCDC68*	3	2.1	3	0.2	6.83×10^{-04}	9.7
78	*CNN2*	3	2.1	3	0.2	6.83×10^{-04}	9.7
79	*ZNF428*	3	2.1	3	0.2	6.83×10^{-04}	9.7
80	*RNF149*	3	2.1	3	0.2	6.83×10^{-04}	9.7
81	*KIAA1715*	3	2.1	3	0.2	6.83×10^{-04}	9.7
82	*DNAJC5G*	3	2.1	3	0.2	6.83×10^{-04}	9.7
83	*LBH*	3	2.1	3	0.2	6.83×10^{-04}	9.7
84	*WDR48*	3	2.1	3	0.2	6.83×10^{-04}	9.7
85	*ZNF879*	3	2.1	3	0.2	6.83×10^{-04}	9.7
86	*MAD2L1BP*	3	2.1	3	0.2	6.83×10^{-04}	9.7
87	*ZNF716*	3	2.1	3	0.2	6.83×10^{-04}	9.7
88	*FOXH1*	3	2.1	3	0.2	6.83×10^{-04}	9.7
89	*IFNA6*	3	2.1	3	0.2	6.83×10^{-04}	9.7
90	*CTR9*	5	3.4	9	0.6	7.58×10^{-04}	5.5
91	*EEF2K*	5	3.4	9	0.6	7.58×10^{-04}	5.5

92	*KDM8*	5	3.4	9	0.6	7.58×10^{-04}	5.5
93	*WDR33*	5	3.4	9	0.6	7.58×10^{-04}	5.5
94	*ITGAV*	5	3.4	9	0.6	7.58×10^{-04}	5.5
95	*KIF15*	8	5.5	21	1.5	7.73×10^{-04}	3.8
96	*PLEKHG4*	7	4.8	17	1.2	9.00×10^{-04}	4.1
97	*DSC3*	7	4.8	17	1.2	9.00×10^{-04}	4.1
98	*MSH2*	7	4.8	17	1.2	9.00×10^{-04}	4.1
99	*DOT1L*	9	6.2	26	1.9	9.05×10^{-04}	3.5
100	*BOC*	9	6.2	26	1.9	9.05×10^{-04}	3.5
101	*LMNTD2*	6	4.1	13	0.9	9.31×10^{-04}	4.6
102	*PRICKLE1*	6	4.1	13	0.9	9.31×10^{-04}	4.6
103	*GPR132*	6	4.1	13	0.9	9.31×10^{-04}	4.6
104	*MISP*	6	4.1	13	0.9	9.31×10^{-04}	4.6
105	*RBM12B*	6	4.1	13	0.9	9.31×10^{-04}	4.6
106	*PDZD7*	4	2.7	6	0.4	9.43×10^{-04}	6.5
107	*MALRD1*	4	2.7	6	0.4	9.43×10^{-04}	6.5
108	*PBLD*	4	2.7	6	0.4	9.43×10^{-04}	6.5
109	*TBRG1*	4	2.7	6	0.4	9.43×10^{-04}	6.5
110	*MRGPRX3*	4	2.7	6	0.4	9.43×10^{-04}	6.5
111	*MPPED2*	4	2.7	6	0.4	9.43×10^{-04}	6.5
112	*NHLRC3*	4	2.7	6	0.4	9.43×10^{-04}	6.5
113	*BACH1*	4	2.7	6	0.4	9.43×10^{-04}	6.5
114	*ACVR2B*	4	2.7	6	0.4	9.43×10^{-04}	6.5
115	*LRRC4*	4	2.7	6	0.4	9.43×10^{-04}	6.5
116	*ABHD11*	4	2.7	6	0.4	9.43×10^{-04}	6.5
117	*APC*	12	8.2	40	2.9	6.56×10^{-04}	3.0

Supplementary Table S4: List of variants significantly related to CRC risk in seven SKAT genes.

S No	Gene	Chr	Position	Ref	Alt	No. of Cases	%	No. of Controls	%	P-value	Odds ratio
1	*GPSM3*	chr6	32,159,931	G	C	7	4.8	0	0.0	0.00×10^{-00}	150.1
2	*TMEM229A*	chr7	123672944	-	GA G	7	4.8	0	0.0	0.00×10^{-00}	150.1
3	*TNXB*	chr6	32,029,249	G	A	3	2.1	0	0.0	8.36×10^{-00}	68.1
4	*ANKRD33B*	chr5	10,638,251	C	T	2	1.4	0	0.0	1.22×10^{-05}	48.3
5	*TNXB*	chr6	32,049,429	G	T	2	1.4	0	0.0	1.22×10^{-05}	48.3
6	*TNXB*	chr6	32,052,363	G	T	2	1.4	0	0.0	1.22×10^{-05}	48.3
7	*TAP2*	chr6	32,800,564	G	C	2	1.4	0	0.0	1.22×10^{-05}	48.3
8	*TAP2*	chr6	32,803,106	G	A	2	1.4	0	0.0	1.22×10^{-05}	48.3
9	*ANKRD33B*	chr5	10,564,604	C	T	1	0.7	0	0.0	0.002	28.8
10	*ANKRD33B*	chr5	10,564,904	G	C	1	0.7	0	0.0	0.002	28.8
11	*ANKRD33B*	chr5	10,649,618	C	T	1	0.7	0	0.0	0.002	28.8
12	*TNXB*	chr6	32,020,522	A	G	1	0.7	0	0.0	0.002	28.8
13	*TNXB*	chr6	32,020,588	G	A	1	0.7	0	0.0	0.002	28.8
14	*TNXB*	chr6	32,030,131	G	T	1	0.7	0	0.0	0.002	28.8
15	*TNXB*	chr6	32,030,189	G	A	1	0.7	0	0.0	0.002	28.8
16	*TNXB*	chr6	32,041,457	G	A	1	0.7	0	0.0	0.002	28.8
17	*TNXB*	chr6	32,041,621	G	A	1	0.7	0	0.0	0.002	28.8
18	*TNXB*	chr6	32,041,679	G	A	1	0.7	0	0.0	0.002	28.8
19	*TNXB*	chr6	32,049,219	C	T	1	0.7	0	0.0	0.002	28.8
20	*TNXB*	chr6	32,064,047	C	T	1	0.7	0	0.0	0.002	28.8
21	*TNXB*	chr6	32,064,464	A	C	1	0.7	0	0.0	0.002	28.8
22	*TNXB*	chr6	32,065,005	T	C	1	0.7	0	0.0	0.002	28.8
23	*TNXB*	chr6	32,065,863	C	T	1	0.7	0	0.0	0.002	28.8
24	*TAP2*	chr6	32,800,549	C	T	1	0.7	0	0.0	0.002	28.8
25	*TAP2*	chr6	32,800,577	C	T	1	0.7	0	0.0	0.002	28.8
26	*TAP2*	chr6	32,803,058	C	T	1	0.7	0	0.0	0.002	28.8
27	*TAP2*	chr6	32,805,387	T	C	1	0.7	0	0.0	0.002	28.8
28	*TMEM229A*	chr7	123,672,509	C	-	1	0.7	0	0.0	0.002	28.8
29	*GPR112*	chrX	135,390,947	A	G	1	0.7	0	0.0	0.002	28.8
30	*GPR112*	chrX	135,405,191	T	C	1	0.7	0	0.0	0.002	28.8
31	*GPR112*	chrX	135,405,471	A	G	1	0.7	0	0.0	0.002	28.8
32	*GPR112*	chrX	135,428,194	A	T	1	0.7	0	0.0	0.002	28.8
33	*GPR112*	chrX	135,431,195	C	A	1	0.7	0	0.0	0.002	28.8
34	*GPR112*	chrX	135,431,514	G	A	1	0.7	0	0.0	0.002	28.8
35	*GPR112*	chrX	135,441,498	C	A	1	0.7	0	0.0	0.002	28.8
36	*ANKRD33B*	chr5	10,649,841	G	T	1	0.7	0	0.0	0.002	28.8
37	*ANKRD33B*	chr5	10,649,842	C	T	1	0.7	0	0.0	0.002	28.8
